# Refilling Prescriptions

**Published:** 1905-12

**Authors:** R. B. Kelley

**Affiliations:** Atlanta, Ga.; Prescriptionist with Frank Edmondson & Bro., 14 South Broad Street


### REFILLING PRESCRIPTIONS.

By R. B. KELLEY, Atlanta, Ga.

Prescriptionist with Frank Edmondson & Bro., 14 South Broad Street.

To discuss this question involves the ownership of the prescription, a subject recently treated very extensively in the pharmaceutical journals. The opinions expressed were at such variance as to preclude all possibility of reaching any definite conclusions, each of the three parties directly interested being advocated as the rightful owner of the prescription. The fact that the physician is the author of the prescription should establish the ownership, but quite a number of reasons testify to the same fact; the physician, being the source of the prescription, emanating as it does from his mind, trained by years of study and experience, and it being absolutely necessary to the success of his practice that he is able to control and direct its administration. The prescription is an instrument in his hands to treat disease. The fact that the patient pays the physician a fee does not imply that he comes into ownership of the prescription, because as long as he keeps the prescription in his possession it can not be of any use to him, but so far as he is concerned it represents an order to the druggist to prepare the treatment desired by the physician, and he has no more right of ownership than he would have to the instruments with which the physician performs an operation, removes a tumor or amputates a limb. He simply pays for the treatment and the result of the treatment. He pays the physician to cure him and has no further right to extend to his friends and acquaintances in having the prescription refilled, after diagnosing their diseases, and he has no right to have the prescription refilled while under the treatment of his physician, unless ordered to do so by the physician, because the physician as a scientific man may wish to give a definite amount of a certain drug to produce a certain effect, when a continued use of the prescription would defeat his purpose and cause confusion in his treatment. And he has no right in a subsequent illness to again refer to this prescription, as the physician may have prescribed for symptoms that he did not mention and of which the patient knew nothing.

The druggist can have no proprietary right to the prescription, as it is merely through courtesy that he is permitted to prepare the medicine, which he has gained for himself with the confidence reposed in him by the physician and the public—a confidence well merited if he has qualified himself for his duties, which require as much intelligence and application as that necessary in therapeutics.

Then who has the right to have the prescription refilled? The physician being the author of it, and intending it for a specific purpose, is naturally the one to control the refilling of the prescription, and it is not to his interest to do so, because it encourages a bad practice and can serve no good purpose to any one. In a case where the physician desires to continue the same treatment, he can easily refer to the prescription (if he has not recorded it in his casebook) by telephoning the druggist. So when we consider the high standard of the scientific physician and pharmacist of today and that diagnosing, prescribing and compounding are based on strictly scientific principles, we must conclude that when the prescription has once been dispensed that it has served the purpose for which it was intended and should be filed exclusively for the physician's reference.

